# Sexual activity and sexual dysfunction in patients with schizophrenia at the Arrazi psychiatric hospital in Salé

**DOI:** 10.1192/j.eurpsy.2024.1535

**Published:** 2024-08-27

**Authors:** N. Ait Bensaid, Y. Bensalah, H. Boukidi, F. El Omari

**Affiliations:** Psychiatric hospital Arrazi, salé, Morocco

## Abstract

**Introduction:**

Sexuality is a natural component of human behavior. Sexual health is “a physical, emotional, mental and social state related to sexuality; it is not merely the absence of disease, dysfunction or infirmity. Sexual dysfunction and poor quality of sex life are common in patients with schizophrenia. The prevalence of sexual dysfunction is higher in people with mental disorders, and may be related to psychopathology and pharmacotherapy.

**Objectives:**

Evaluate sexual activity, sexual dysfunction and its consequences in patients with schizophrenia followed and hospitalized in the various structures of the Arrazi psychiatric hospital in Salé.

**Methods:**

This is a descriptive cross-sectional study using a questionnaire including sociodemographic and clinical criteria, data on sexual behavior and the Arizona Sexual Experience Scale (ASEX) to assess sexual activity, sexual dysfunction and its consequences in patients with schizophrenia followed and hospitalized in the various structures of the Arrazi psychiatric hospital in Salé. Inclusion criteria: patients of both sexes diagnosed with schizophrenia according to DSM 5 criteria, age greater than or equal to 20 years. Exclusion criteria: intellectual disability, general medical condition known to cause sexual dysfunction (diabetes mellitus, history of vascular accident, congestive heart failure, unstable heart condition, arrhythmia or myocardial infarction in the last six months).

**Results:**

We collected 157 participants. 81% of the participants were men, 67% of whom had left school at college. The majority of patients were born in the city. 85% were unemployed. 89% were heterosexual and 77% were single. 92% smoked cigarettes. 66% had schizophrenia for more than 5 years with 55% having poor adherence to antipsychotics with around 65% on atypical antipsychotics. Around 42% reported currently having sexual relations. 56% of participants had sexual dysfunction, and 67% were dissatisfied with the quality of their sexual relations.

**Conclusions:**

Sexual dysfunction is prevalent in schizophrenic patients, and these problems can be linked to both the illness and its treatment. Sexual dysfunction is also an important factor in therapeutic compliance, which is strongly influenced by the side effects of antipsychotics. It is therefore necessary to know more about the sexual side-effects of medication on patients, and doctors should also systematically ask patients about their sexual history before prescribing psychotropic drugs.

**Disclosure of Interest:**

None Declared

